# The role of l-leucovorin uptake and metabolism in the modulation of 5-fluorouracil efficacy and antifolate toxicity

**DOI:** 10.3389/fphar.2024.1450418

**Published:** 2024-08-21

**Authors:** Godefridus J. Peters, Ietje Kathmann, Elisa Giovannetti, Kees Smid, Yehuda G. Assaraf, Gerrit Jansen

**Affiliations:** ^1^ Department of Medical Oncology, Cancer Center Amsterdam, Amsterdam University Medical Centers, Vrije Universiteit Amsterdam, Amsterdam, Netherlands; ^2^ Department of Biochemistry, Medical University of Gdansk, Gdansk, Poland; ^3^ Cancer Pharmacology Lab, Fondazione Pisana per la Scienza, Pisa, Italy; ^4^ The Fred Wyszkowski Cancer Research Laboratory, Faculty of Biology, The Technion-Israel Institute of Technology, Haifa, Israel; ^5^ Amsterdam Rheumatology and Immunology Center, Amsterdam University Medical Centers, Vrije Universiteit Amsterdam, Amsterdam, Netherlands

**Keywords:** leucovorin stereo-isomers, l-leucovorin, thymidylate synthase, 5-fluorouracil, antifolates, pemetrexed

## Abstract

**Background:**

L-Leucovorin (l-LV; 5-formyltetrahydrofolate, folinic acid) is a precursor for 5,10-methylenetetrahydrofolate (5,10-CH_2_-THF), which is important for the potentiation of the antitumor activity of 5-fluorouracil (5FU). LV is also used to rescue antifolate toxicity. LV is commonly administered as a racemic mixture of its l-LV and d-LV stereoisomers. We compared dl-LV with l-LV and investigated whether d-LV would interfere with the activity of l-LV.

**Methods:**

Using radioactive substrates, we characterized the transport properties of l-LV and d-LV, and compared the efficacy of l-LV with d-LV to potentiate 5FU-mediated thymidylate synthase (TS) inhibition. Using proliferation assays, we investigated their potential to protect cancer cells from cytotoxicity of the antifolates methotrexate, pemetrexed (Alimta), raltitrexed (Tomudex) and pralatrexate (Folotyn).

**Results:**

l-LV displayed an 8-fold and 3.5-fold higher substrate affinity than d-LV for the reduced folate carrier (RFC/SLC19A1) and proton coupled folate transporter (PCFT/SLC46A1), respectively. In selected colon cancer cell lines, the greatest enhanced efficacy of 5FU was observed for l-LV (2-fold) followed by the racemic mixture, whereas d-LV was ineffective. The cytotoxicity of antifolates in lymphoma and various solid tumor cell lines could be protected very efficiently by l-LV but not by d-LV. This protective effect of l-LV was dependent on cellular RFC expression as corroborated in RFC/PCFT-knockout and RFC/PCFT-transfected cells. Assessment of TS activity *in situ* showed that TS inhibition by 5FU could be enhanced by l-LV and dl-LV and only partially by d-LV. However, protection from inhibition by various antifolates was solely achieved by l-LV and dl-LV.

**Conclusion:**

In general l-LV acts similar to the dl-LV formulations, however disparate effects were observed when d-LV and l-LV were used in combination, conceivably by d-LV affecting (anti)folate transport and intracellular metabolism.

## 1 Introduction

L-Leucovorin (l-LV; 5-formyltetrahydrofolate; (6S)-leucovorin) is a precursor for natural reduced folates and is widely used, either in its pure form or in a racemic mixture with d-leucovorin (d-LV; (6R)-leucovorin), in combination treatment of colorectal cancer with 5-fluorouracil (5FU) (Thirion et al., 2004; Van Cutsem et al., 2011; Van Cutsem et al., 2016; Yoshino et al., 2018; Glimelius et al., 2021). As the active isomer, l-LV has partially replaced the use of the racemic mixture of d- and l-LV in a number of treatment schedules for colorectal cancer (Kovoor et al., 2009; Baumgaertner et al., 2010; Danenberg et al., 2016). L-LV may also be used as rescue regimen for the treatment with methotrexate (MTX), an antifolate drug which, in a high-dose regimen is widely used for the treatment of leukemia and osteosarcoma (Walling, 2006). Because of the shortage of chemotherapeutics, including dl-LV, interests in alternatives including l-LV (marketed as Fusilev^®^/levoleucovorin) has renewed research regarding its optimal administration in the treatment of these malignancies (Kovoor et al., 2009; Fujii et al., 2011; Chuang and Suno, 2012; Hayes et al., 2014; Danenberg et al., 2016). Although it was also suggested that the oxidized folic acid might replace LV, folic acid does not modulate 5FU activity (Asbury et al., 1987). L-LV enhances the antitumor activity of 5FU by increasing and prolonging the inhibition of thymidylate synthase (TS) (Peters et al., 2002; Danenberg et al., 2016). TS is inhibited by 5FU via its metabolite 5-fluoro-2′-deoxy-5′-uridinemonophosphate (FdUMP) (Peters et al., 2002; Longley et al., 2003) which, in the absence of reduced folate cofactors, forms an unstable binary covalent complex with TS. Upon administration of l-LV, a stable ternary covalent complex will be formed with the l-LV metabolite 5,10-methylenetetrahydrofolate (5,10-CH_2_-THF). In contrast to the binary complex, this ternary complex is very stable and inhibition of TS in patients will be retained for several days; the duration of this TS inhibition is dependent on the biochemical properties/expression dynamics of TS of individual patients (Peters et al., 2002). In fact, treatment with either a conventional bolus of 5FU or with a continuous infusion (either 1 week or longer) can induce the expression of TS up to 3-5-fold (Peters et al., 1993; Codacci-Pisanelli et al., 1995). Notably, pre- and simultaneous treatment with LV, as well as a high dose 5FU (with uridine protection) can abolish the induction of TS (Codacci-Pisanelli et al., 1997; Codacci-Pisanelli et al., 2002; Peters et al., 2002). This can partially be explained by the findings of Chu et al. (1991), who originally discovered an autoregulatory loop in which binding of thymidylate synthase protein to its own mRNA regulates its translation, while 5,10-CH_2_-THF relieves this effect. However, Kitchens et al. (1999) postulated that 5FU treatment leads to a FdUMP-mediated stabilization of the TS protein, leading to 5FU resistance. Previously we also observed in patients that 5FU induced increased TS protein and enzyme activity levels, but not TS mRNA, while LV treatment prevented the increase in TS levels (Peters et al., 2002). Apparently, a high-dose of 5FU has a similar effect. The FOLFOX and FOLFIRI schedules combine the advantages of both a high bolus dose and an infusion of 1–2 days at a relatively high dose (Van Cutsem et al., 2016; Glimelius et al., 2021).

**FIGURE 1:**
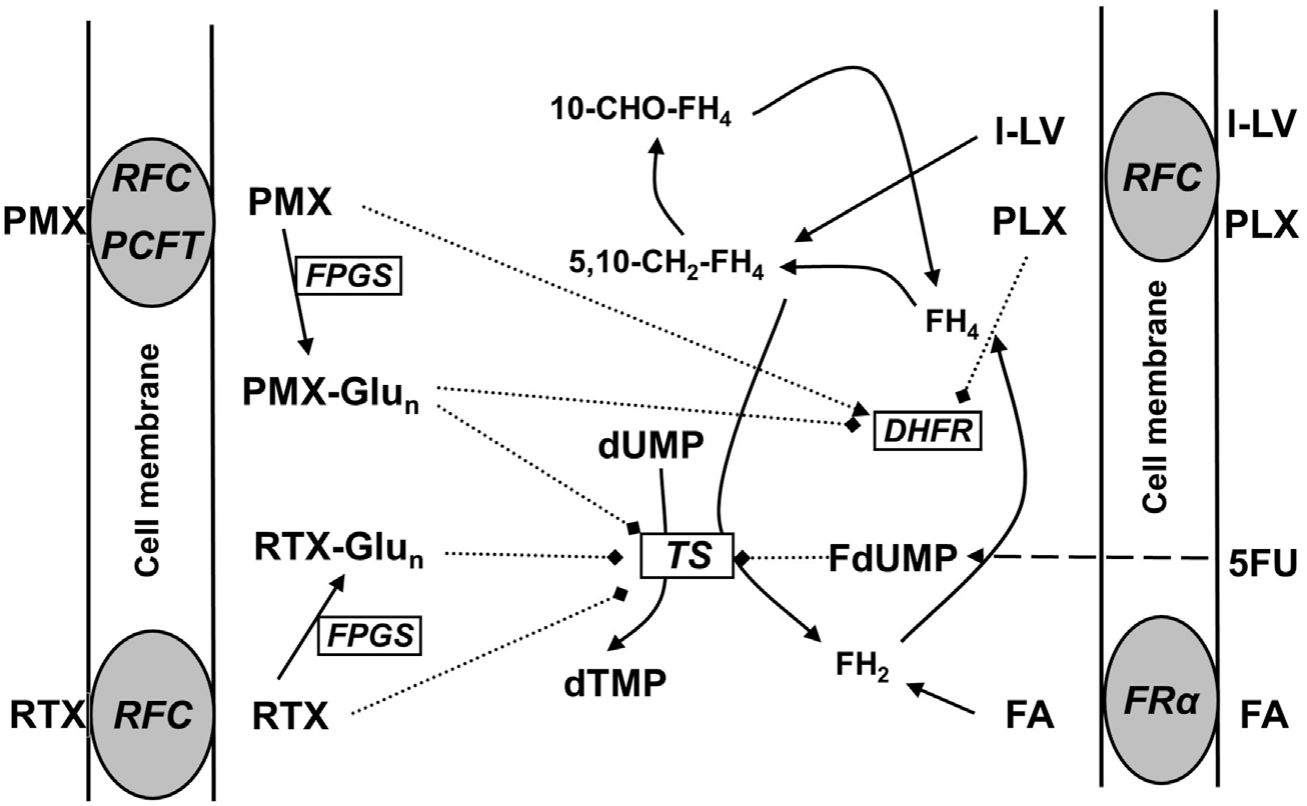
Schematic overview of folate transporters in cancer cells and the mechanism of action of the antifolates pralatrexate (PLX), pemetrexed (PMX), and raltitrexed (RTX). PCFT, at the optimal pH of 5.5, is the major transporter for PMX, RTX is primarily transported by the RFC, and FA by FRα. FA and l-LV may also be transported by PCFT, especially in the gut. Intracellularly all (anti) folates (including l-LV and PLX, not depicted) are metabolized via polyglutamylation; the polyglutamylated forms of the antifolates PMX and RTX can (more potently) inhibit DHFR and/or TS, while FA and l-LV enter the folate cycle. L-LV is metabolized to 5,10-methylene-tetrahydrofolate (5,10-CH_2_-FH_4_). After cellular uptake 5FU is metabolized via several steps to FdUMP, which is a suicide inhibitor of TS by forming a ternary complex with TS and 5,10-CH_2_-FH_4._

In addition to the potentiation of 5FU-mediated TS inhibition, LV can also protect cells against the toxicity of antifolates (Walling, 2006) such as the dihydrofolate reductase (DHFR) inhibitors methotrexate (MTX) (Bertino, 1993), PLX (Folotyn) (Azzoli et al., 2007) and other antifolates such as the folate-based TS inhibitors pemetrexed (PMX, ALIMTA) and raltitrexed (RTX, Tomudex) (Figure 1). PMX is registered for the treatment of non-squamous non-small cell lung cancer (NSCLC) (Gridelli et al., 2010) and malignant pleural mesothelioma (MPM); it is usually given in combination with a platinum drug, either cisplatin or carboplatin (Vogelzang et al., 2003; Galvani et al., 2011). RTX is registered for the treatment of colon cancer, but has never become a standard treatment option (Van Cutsem et al., 2002; Batra et al., 2021). PLX is registered for the treatment of peripheral T-cell lymphoma (PTCL) and diffuse B-cell lymphoma (DBCL) (Azzoli et al., 2007; O’Connor et al., 2011; Peters et al., 2020). Although other natural folates such as folic acid and 5-methyltetrahydrofolate (5-CH_3_-THF) can also protect cells from antifolate cytotoxicity (Dudman et al., 1982; Reggev and Djerassi, 1986; Westerhof et al., 1995a), LV is much more stable than 5-CH_3_-THF and the activity of l-LV is instantaneous and more effective, and lower concentrations are required. This prompt effect is partly due to the efficient uptake into cells predominantly mediated by the reduced folate carrier (RFC/SLC19A1) (Matherly and Hou, 2008; Zhao et al., 2009; Gonen and Assaraf, 2012; Frigerio et al., 2019). In contrast, in cancer cells, folic acid is preferentially taken up by a folate receptor (FR) which has a high affinity, but a low capacity (Nutt et al., 2010; Assaraf et al., 2014), while intestinal uptake is predominantly mediated by the proton-coupled folate transporter (PCFT/SLC46A1) (Zhao et al., 2008; Zhao et al., 2009; Matherly et al., 2018). Based on these features, LV is used to prevent the side effects of MTX treatment in cancer patients. LV is usually given 24 h after treatment with high-dose MTX when drug levels are higher than 1 µM (Relling et al., 1994).

LV is widely used in the treatment of colon cancer in regimens such as the weekly and monthly 5FU-LV regimens (Roswell Park and Mayo regimens, respectively) (Piedbois et al., 1992; Thirion et al., 2004; Van Cutsem et al., 2016), but currently mostly in combination with oxaliplatin or irinotecan in the FOLFOX and FOLFIRI regimens, respectively (Rougier and Lepère, 2005; Van Cutsem et al., 2016). In pancreatic cancer, FOLFIRINOX (a combination of FOLFOX and FOLFIRI) is an active regimen, although at the expense of increased toxicity (Conroy et al., 2011; Caparello et al., 2016). Most of these combinations have been investigated and optimized for the racemic mixture of d- and l-LV. Despite decades of research on folate metabolism, a number of questions remain to be addressed regarding the action of l-LV and the interaction with these drugs. This is, among other factors, due to the fact that novel transporters (e.g., PCFT, and efflux pumps such as the ABC transporters MRP1-5 and BCRP) have been recognized to play a role in influx and efflux of both natural folates and antifolates (Hooijberg et al., 1999; Hooijberg et al., 2004; Shafran et al., 2005; Assaraf, 2006; Lemos et al., 2009; Gonen and Assaraf, 2012; Matherly et al., 2018; Hooijberg et al., 2014).

Although d-LV may be taken up into cells via the normal folate transport systems albeit at a lower efficiency than l-LV, and is not metabolized or less efficiently than l-LV, it has been reported to interfere with the pharmacokinetics of l-LV (Sirotnak et al., 1979; Zittoun et al., 1993) and may have some antitumor effect by itself (Carlsson et al., 1995). Following some discussions on drug shortage (Chuang and Suno, 2012) it was suggested that l-LV could be replaced by folic acid, the oxidized form of folate, despite early studies showing that it is ineffective in modulation of 5FU (Asbury et al., 1987). However, intestinal uptake of folic acid is predominantly mediated by PCFT and to a lesser extent by FR (Matherly and Hou, 2008; Zhao et al., 2008; Nutt et al., 2010). Subsequently it needs to be activated by DHFR to produce the active reduced folate forms dihydrofolate (DHF) and tetrahydrofolate (THF). DHFR in humans has a markedly lower capacity (50-fold) to convert folic acid to DHF and THF then in widely used animal model systems such as the mouse (Bailey and Ayling, 2009). In humans, this will limit its metabolism and lead to a larger fraction of unmetabolized folic acid in the circulation of patients receiving high doses of folic acid (>1 mg per day) (Maruvada et al., 2020). Based on these considerations we investigated the uptake, metabolism and efflux of l-LV in relevant model systems, the potential interaction with d-LV, and the impact of these LV formulations on the sensitivity to several antifolates and 5FU.

## 2 Methods

### 2.1 Materials

RPMI-1640 medium without folic acid (LF) was purchased from Invitrogen. Standard RPMI-1640 medium and DMEM medium, trypsin/EDTA (170,000 Units trypsin/L; 200 mg EDTA/L), Fetal Bovine Serum, dialyzed Fetal Bovine Serum, penicillin/streptomycin (100,000 IU/mL) and Hank’s Balanced Salt Solution (HBSS) were purchased from Lonza (Lonza, Geleen, Netherlands). Cell culture flasks were purchased from Greiner Bio one (Alphen aan de Rijn, Netherlands). L-LV (Fusilev^®^) and pralatrexate (Folotyn^®^) were gifts from Spectrum Pharmaceuticals (currently licensed to Acrotech, East Windsor, NJ, United States). d-LV, dl-LV, 5-FU and sulforhodamine B (SRB) were purchased from Sigma-Aldrich (Amsterdam, the Netherlands). Pemetrexed was a gift from Eli Lilly and Company (Indianapolis, IN, United States), and Raltitrexed from Astra-Zeneca (Cambridge, United Kingdom). The antifolates were dissolved in 150 mM NaHCO_3_ to stock solutions of 10 mM and stored at −20°C until use. Drug dilutions were made in medium prior to experiments. Trizol and RT-qPCR kit were from Invitrogen/Thermo Fisher Scientific (Bleiswijk, the Netherlands). Radioactive compounds, [3′,5′,7,9-^3^H(N)]-(6S)-Leucovorin (25 Ci/mmol), [3′,5′,7,9-^3^H]-folic acid, diammonium salt (21.0 Ci/mmol) and [5-^3^H(N)]-2′-deoxycytidine (25 Ci/mmol) were obtained from Moravek Biochemicals (Brea, CA, United States).

### 2.2 Model systems

In order to investigate the role of l-LV in the potentiation of 5FU we employed a panel of colon cancer cell lines, previously characterized for their sensitivity to 5FU and modulation by l-LV or dl-LV (Van Triest et al., 1999). We selected cell lines based on their variation in 5FU sensitivity, while we added cell lines which were adapted to grow under low folate concentrations, hence better representing the physiological folate environment (Lemos et al., 2008; Lemos et al., 2009). In order to investigate the role of various transporters in the uptake of l-LV and antifolates and their role in protection against cytotoxicity of several antifolates we used several cell lines, either deficient in one of the transporters or having an overexpression of one of the transporters, as well as mesothelioma and NSCLC cell lines previously characterized for their sensitivity to antifolates (Backus et al., 2000; Giovannetti et al., 2008; Giovannetti et al., 2017) (Table 1).

**TABLE 1:** Characteristics of cell lines.

Cell lines	Source	Medium	Serum (FBS)	Remarks
WiDr	Colon cancer	RPMI 1640	10% FBS	
WiDr LF/LV	Colon cancer	RPMI 1640 LF	10% dFBS	2.5 nM l-LV
CaCo2	Colon cancer	DMEM	10% FBS	
CaCo2 LF/LV	Colon cancer	RPMI 1640 LF	10% dFBS	2.5 nM l-LV
CaCo2-LF/FA	Colon cancer	RPMI 1640 LF	10% dFBS	2.5 nM FA
LS174T	Colon cancer	DMEM	10% FBS	
HT29	Colon cancer	DMEM	10% FBS	
SW948	Colon cancer	DMEM	10% FBS	
SW1398	Colon cancer	DMEM	10% FBS	
NCI-H28	MPM	RPMI 1640	20% FBS	
MSTO-211H	MPM	RPMI 1640	10% FBS	
NCI-H292	NSCLC	RPMI 1640	10% FBS	
NCI-H460	NSCLC	RPMI 1640	10% FBS	
CCRF-CEM	ALL	RPMI 1640	10% FBS	RFC+
CEM/MTX	CEM variant	RPMI 1640	10% FBS	RFC−
CEM-7A	CEM variant	RPMI 1640-LF	10% dFBS (0.25 nM l-LV)	RFC+++ (30-fold)
AA8	CHO wild type	RPMI 1640	10% FBS	RFC++/PCFT−
CHO/C5	CHO k.o.	RPMI 1640	10% FBS	RFC−/PCFT−
CHO/C5-Mock	CHO	RPMI 1640	10% FBS	RFC−/PCFT−
CHO/C5-hRFC	CHO	RPMI 1640 LF	10% dFBS (1 nM l-LV)	RFC++/PCFT−
CHO/C5-PCFT	CHO	RPMI 1640	10% FBS	RFC−/PCFT++
KB	Nasopharyngeal epidermoid	RPMI 1640-LF	10% FBS	FRα++

l-LV, l-leucovorin; FA, folic acid; FBS, fetal bovine serum; dFBS: dialyzed fetal bovine serum; MPM, malignant pleural mesothelioma; NSCLC, non-small cell lung cancer; ALL, acute lymphoblastic leukemia; CHO, Chinese hamster ovary; CHO/C5, AA8 cells in which the natural RFC has been knocked out (k.o.), while the cells do not have natural PCFT; CHO/C5 mock, cells transfected with an empty vector; LF, low folate; HF, high folate; RFC-, RFC-deficient; RFC+++, RFC overexpression; PCFT−, PCFT-deficient; PCFT++, PCFT overexpression; FRα−, FRα deficient; FRα++, overexpression. Additional details and sources of cell lines are described in (Jansen et al., 1990; 1997; Van Triest et al., 1999; Backus et al., 2000; Cillessen et al., 2008; Giovannetti et al., 2008; 2017; Lasry et al., 2008; Lemos et al., 2008; Lemos et al., 2009).

### 2.3 Cell culture

Most culture conditions are described in the papers cited in the legend of Table 1. Briefly, solid tumor cell lines were maintained as monolayer cultures either in DMEM (containing 2 mM L-glutamine and a supra-physiological concentration of 8.8 µM folic acid), RPMI-1640 (containing 2.3 μM supra-physiological folic acid (FA) and 2 mM L-glutamine), or RPMI-1640 (without FA, low folate (LF)). Both DMEM and RPMI-1640 (HF) were supplemented with 10% heat-inactivated fetal bovine serum (FBS) and 20 mM Hepes pH 7.4 including 1% penicillin/streptomycin (100, IU/mL). RPMI-1640 LF medium was supplemented with 10% dialysed fetal bovine serum and 20 mM Hepes pH 7.4. Cells originating from the CaCo-2 and WiDr cell lines were adapted to grow in RPMI-1640 LF medium supplemented with 2.5 nM l-LV as a representative for a more physiological environment (Backus et al., 2000; Lemos et al., 2008). Cells were grown in 25 cm^2^ and 75 cm^2^ flasks in a 37°C incubator with 5% CO_2_ and 100% humidity. Cells were harvested with trypsin/EDTA at a point of 80% confluency in their exponential growth.

### 2.4 Growth inhibition experiments

Growth inhibition of the non-small cell lung cancer (NSCLC), colon cancer and malignant pleural mesothelioma (MPM) cell lines by 5-FU and the modulation by l-LV, d-LV and dl-LV, growth inhibition by PMX, RTX and PLX and the protection by l-LV was determined using the sulforhodamine B (SRB) assay as previously described (Keepers et al., 1991). Briefly, cells were seeded in triplicate in 96-well plates at their pre-established optimal density (CHO cells: 2,500 cells/100 μL; WiDr and HT29: 3,500 cells/100 μL; WiDr-LF/LV, LS174T: 5,000 cells/100 μL; CaCo2, CaCo2-LF/LV, H292, H28 and MSTO-211H: 6,000 cells/100 μL; H460, SW948 and SW1398: 7,000 cells/100 μL) and were allowed to attach for ∼ 24 h at 37°C in 5% CO_2_ and 100% humidified incubator. Thereafter, 100 μL of drug dilution without LV, or with 5 μM l-LV/5 μM d-LV/10 μM dL-LV (or any LV alone) were added. Cells were allowed to grow for 72 h at 37°C in 5% CO_2_ and 100% humidity. The t = 0 h control plate consisted of cells plated on the same day as the other cells, but after 24 h at 37°C in 5% CO_2_ and 100% humidity, only 100 μL medium was added and the cells were fixed immediately with 50% trichloroacetic acid (TCA) in milliQ water (25 μL per well) and put at 4°C for at least 1 h. Plates were washed five times with water and dried. Cells were stained with the SRB protein dye (0.4% (w/v) in 1% acetic acid. Excess dye was washed away four times with 1% acetic acid and plates were dried again. 200 μL of Tris (10 mM Tris (hydroxymethyl)-aminomethane in MQ) was added and optical density (OD) was read at 492 nm after mixing 2–3 min on a plate shaker.

Growth inhibition of lymphoma cells was determined using the tetrazolium (MTT) assay, as described earlier (Peters et al., 2020), including the t = 0 and t = 72 h controls.

### 2.5 TS *in situ* inhibition assay (TSIA)

To study the ability of the various LV formulations to modulate drug activity at the molecular target, i.e., TS, the TSIA assay was used (Peters et al., 1999). The principle of this assay is the measurement of TS enzymatic activity in intact cells. Intact cells will readily internalize radioactively labelled [5-^3^H]-deoxycytidine, which will be rapidly converted by deoxycytidine kinase (dCK) to [5-^3^H]-dCMP and subsequently deaminated to [5-^3^H]-dUMP, which will then serve as a substrate in the TS catalysed reaction to dTMP and ^3^H_2_O; the quantity of ^3^H_2_O formed over time is a measure of intracellular TS activity. We earlier showed that [5-^3^H]-deoxycytidine performed better than [5-^3^H]-deoxyuridine, although the latter is a direct substrate for [5-^3^H]-dUMP (Rots et al., 1999). This is because dCK has a higher, cell cycle independent activity than thymidine kinase 1, which is a cell cycle dependent enzyme. For this purpose, cancer cells were cultured in six wells plates at 2.5 × 10^5^ cells/2 mL/well for 24 h. Thereafter, cells were exposed to drugs with or without the LV formulations at the indicated concentrations for 4 h. 2 h before the end of the incubation period [5-^3^H]-2′-deoxycytidine (final concentration 1 µM) was added to the cells. A 200 µL sample of the culture medium was taken and the reaction was terminated by addition of TCA (final concentration 5%) and unconverted [5-^3^H]-deoxycytidine was removed by precipitation with activated charcoal as described earlier (Peters et al., 1999; Rots et al., 1999). ^3^H_2_O was measured with liquid scintillation counting. In most cell lines we quantified dCK activity and dCK mRNA levels. Even in cells with a low dCK activity (Van der Wilt et al., 2003), we observed a relatively high tritiated water signal, not dependent on dCK expression. Therefore, we concluded that dCK activity is not rate-limiting for the TSIA assay (Rots et al., 1999; Van der Wilt et al., 1999).

### 2.6 Receptor binding and transport studies

In order to determine whether the LV isomers were substrates for either folate receptor α (FRα), RFC and/or PCFT, we used model systems with established overexpression of these receptors or transporters (Westerhof et al., 1995a; Westerhof et al., 1995b; Lasry et al., 2008; Van der Heijden et al., 2009).

For FRα we used KB cells (Westerhof et al., 1995b). An intact cell binding assay for competitive binding was performed as described previously (Westerhof et al., 1995a). Briefly, cells were washed with Hanks balanced salt solution and 6 mM glucose (HBSS, pH 7.4), suspended in 1 mL of this solution (1*10^6^ for KB cells) and incubated at 4°C for 15 min with 100 pmol [^3^H]-folic acid [(^3^H)-FA], in the presence and absence of unlabelled folic acid or LV stereoisomers. Cells were collected by centrifugation and analysed for radioactivity. Relative affinities for FRα were defined as the inverse ratio of compound to displace 50% of radioactive folic acid from FRα. The relative affinity of folic acid was set at 1.

CEM-7A cells, which display 30-fold RFC overexpression relative to wild-type CCRF-CEM cells, were used to determine the relative affinity for RFC of the various LV formulations in comparison to MTX as a prototypical RFC substrate (Jansen et al., 1990; Westerhof et al., 1995a). CEM-7A cells (2 × 10^6^) were suspended in 1 mL HBSS and the assay was initiated by addition of 25 μL 200 μM [^3^H]-l-LV; 5,000 pmol (i.e., 5 μM final concentration). Incubations proceeded for 2 min at 37°C in the absence and presence of increasing amounts of unlabelled LV/antifolate drug. Blanks consisted of a separate incubation at 4°C or incubation with 100-fold molar excess of unlabelled l-LV. [^3^H]-l-LV influx was terminated by adding 9 mL ice-cold HBSS, centrifugation and an additional wash of the cells with 10 mL ice-cold buffer. Cell pellets were then assessed for radioactivity. Relative affinities for RFC are expressed as the concentration of unlabelled drug necessary to inhibit [^3^H]-l-LV uptake by 50%.

The substrate affinity of PCFT for the various LV formulations was assessed relative to the antifolate substrate Pemetrexed (PMX) in CHO-C5 cells transfected with human PCFT. CHO/C5 cells were genetically modified to be deficient in RFC activity, and do not express PCFT (Lasry et al., 2008). The competitive inhibition by 2.5 μM [^3^H]-l-LV influx of PCFT was measured essentially as described for the RFC assay, except that the incubation time was 3 min, at pH 5.5 (the optimal pH for PCFT) and pH 7.4 with increasing amounts of unlabelled LV stereoisomers/antifolate drug. For PCFT too, relative affinities were expressed as the concentration of unlabelled drug necessary to inhibit 50% of [^3^H]-l-LV influx.

### 2.7 RT-qPCR

Cells were harvested by trypsinization (trypsin/EDTA), suspended in 5 mL medium and centrifuged for 5 min at 1,500 rpm at 4°C. The cell pellet was suspended in PBS (2 × 10^6^ cells/mL) and 1 mL suspension was transferred into Eppendorf vials and centrifuged again. The supernatant was aspirated and the Eppendorf vials were snap frozen in liquid nitrogen and stored at −80°C. RNA was extracted from cell pellets by adding Trizol (1 mL per 5–10 × 10^6^ cells). RNA was then reverse-transcribed for RT-PCR analysis, which was performed as described previously (Giovannetti et al., 2017).

### 2.8 Statistical analysis

Experiments were performed at least in triplicate. Data were expressed as mean ± SEM and analyzed by a t-test, Pearson correlation or two-way analysis of variance (2-way ANOVA) by GraphPad Prism software, version 5.1. Two-way ANOVA was further analysed using Tukey’s multiple comparison test. Level of significance is *p* < 0.05, if not otherwise stated.

## 3 Results

### 3.1 The role of folate transporters in the intracellular uptake of -LV isomers

**FIGURE 2:**
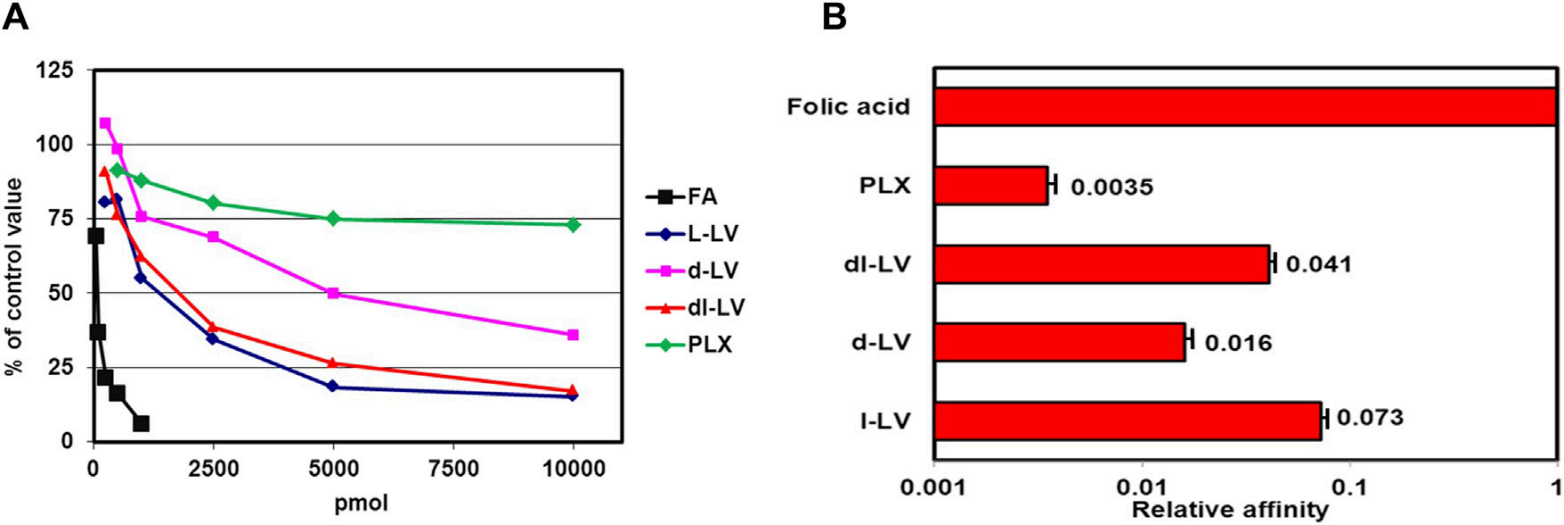
Competitive displacement of [^3^H]-FA binding from FRα (KB cells) by LV-stereoisomers. **(A)** Intact KB cells were incubated with 100 pmol [^3^H]-FA and in the absence or presence of increasing concentrations unlabeled LV-stereoisomers and pralatrexate (PLX). Concentration- dependent displacement of [^3^H]FA binding is depicted. Representative figure from three separate experiments (SEM was within the size of the marker). **(B)** Relative affinities of FRα for LV-stereoisomers and PLX. Relative affinity is defined as the inverse ratio of the amount of drug displacing 50% of [^3^H]-FA. Relative affinity for FA is set to 1. Values are means ± SEM from three separate experiments.

The relative substrate/binding affinities of the LV stereoisomers were examined for each of the three major folate transporters in cancer cells; RFC, PCFT, and FRα (Figure 1). Displacement of [^3^H]-FA binding from FRα by l-LV and d-LV required 15-fold and 69-fold molar excess over folic acid, respectively (Figure 2A). 50% [^3^H]-FA displacement by dl-LV occurred at 2-fold lower concentrations than l-LV, whereas displacement by PLX was observed at >100-fold molar excess (Figure 2A). Representation as relative affinities for FRα relative to FA showed l-LV: 0.073, dl-LV: 0.041, d-LV: 0.016, and PLX: 0.0035 (Figure 2B).

**FIGURE 3:**
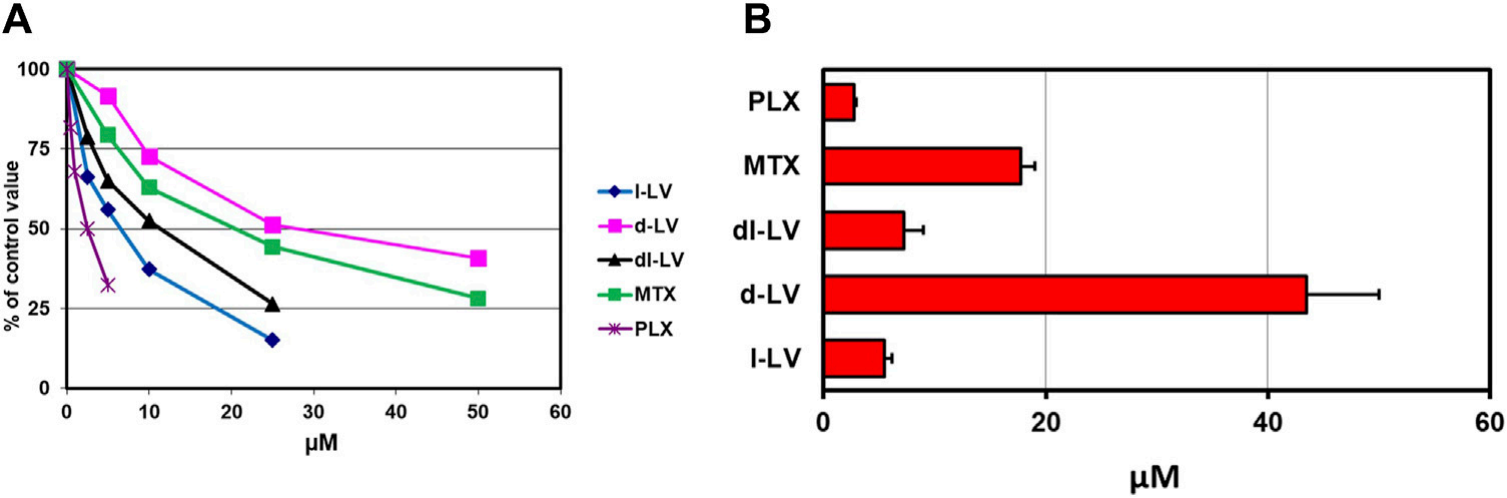
Relative RFC substrate affinity for LV-stereoisomers, PLX and MTX. **(A)** Concentration-dependent inhibition of the RFC-mediated influx of 5 µM [^3^H]-l-leucovorin in CEM 7A cells (with 30-fold overexpression of RFC compared to wild type CCRF-CEM cells) by LV-stereoisomers, PLX and MTX. Representative figure out of three separate experiments is shown (SEM was within the size of the marker). **(B)** 50% [^3^H]-l-LV influx inhibitory concentrations of the LV-stereoisomers, PLX and MTX. Means ± SEM from three separate experiments.

The RFC is a high affinity but low capacity transporter which is responsible for transport of most natural folates under physiological conditions (Jansen et al., 1997; Zhao et al., 2009; Gonen and Assaraf, 2012). Relative affinities of RFC for LV-stereosiomers were assessed by [^3^H]-l-LV influx competition with unlabeled compounds and the antifolates MTX and PLX as reference. Influx competition profiles, at 5 µM [^3^H]-l-LV extracellular concentrations, are shown in Figure 3A. 50% inhibition of [^3^H]-l-LV influx was observed at 5.5 µM l-LV, 7.3 µM dl-LV, and 44 μM d-LV, indicating an 8-fold higher RFC substrate affinity of l-LV over d-LV (Figure 3B). 50% inhibition of [^3^H]-l-LV influx by PLX and MTX was noted at 2.5-fold lower and 3-fold higher concentrations than l-LV, respectively (Figure 3).

**FIGURE 4:**
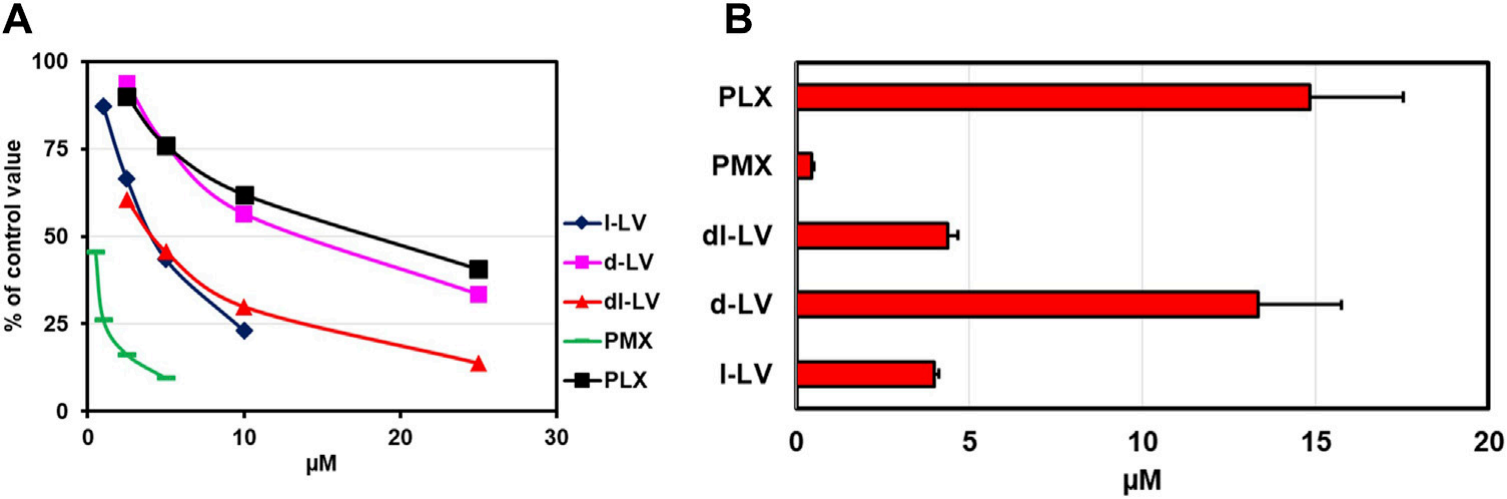
Relative PCFT substrate affinity for LV-stereoisomers, PMX and PLX. **(A)** Concentration-dependent inhibition of PCFT-mediated influx of [^3^H]-l-leucovorin (2.5 µM, CHO/C5/PCFT cells, pH 5.5) by LV-stereoisomers, PMX and PLX. Representative figure out of three separate experiments is shown (SEM was within the size of the marker). **(B)** Compound concentrations (µM) establishing 50% inhibition of [^3^H]-l-LV influx. Values are means ± SEM from three separate experiments.

PCFT is characterized as an (anti)folate transporter with a low pH optimum of around 5.5 (Zhao et al., 2008; Zhao et al., 2009; Matherly et al., 2018). To this end, PCFT influx competition of [^3^H]-l-LV with LV-stereoisomers, the prototypical PCFT substrate PMX, and PLX was measured both at pH 5.5 and 7.4 in CHO/C5/PCFT cells, being RFC-deficient and transfected with PCFT (Lasry et al., 2008). At pH 7.4 and at an extracellular concention of 2.5 µM [^3^H]-l-LV, influx rates were 11% compared to pH 5.5 (Supplementary Figure S1; white bar). At pH 5.5, only 0.4 µM PMX was required to achieve a 50% inhibition of PCFT-mediated [^3^H]-l-LV influx, consistent with its excellent substrate specificity at acidic pH (Zhao et al., 2008) (Figure 4A). 50% inhibition of [^3^H]-l-LV influx was achieved by 4 µM l-LV and by 13.3 µM d-LV (Figure 4B). With a 50% inhibitory concentration of 4.5 µM, dl-LV seemed to be more effective than the sum of the two isomers. Of further note, at pH 7.4, the effect of the drugs was much less than at pH 5.5, suggesting different transporter kinetics at each pH (Supplementary Figure S1).

Together, these findings show that the three main folate transport systems harbor distinct substrate affinities for LV stereoisomers and various antifolates.

### 3.2 RFC and PCFT expression in cancer cell line models

**FIGURE 5:**
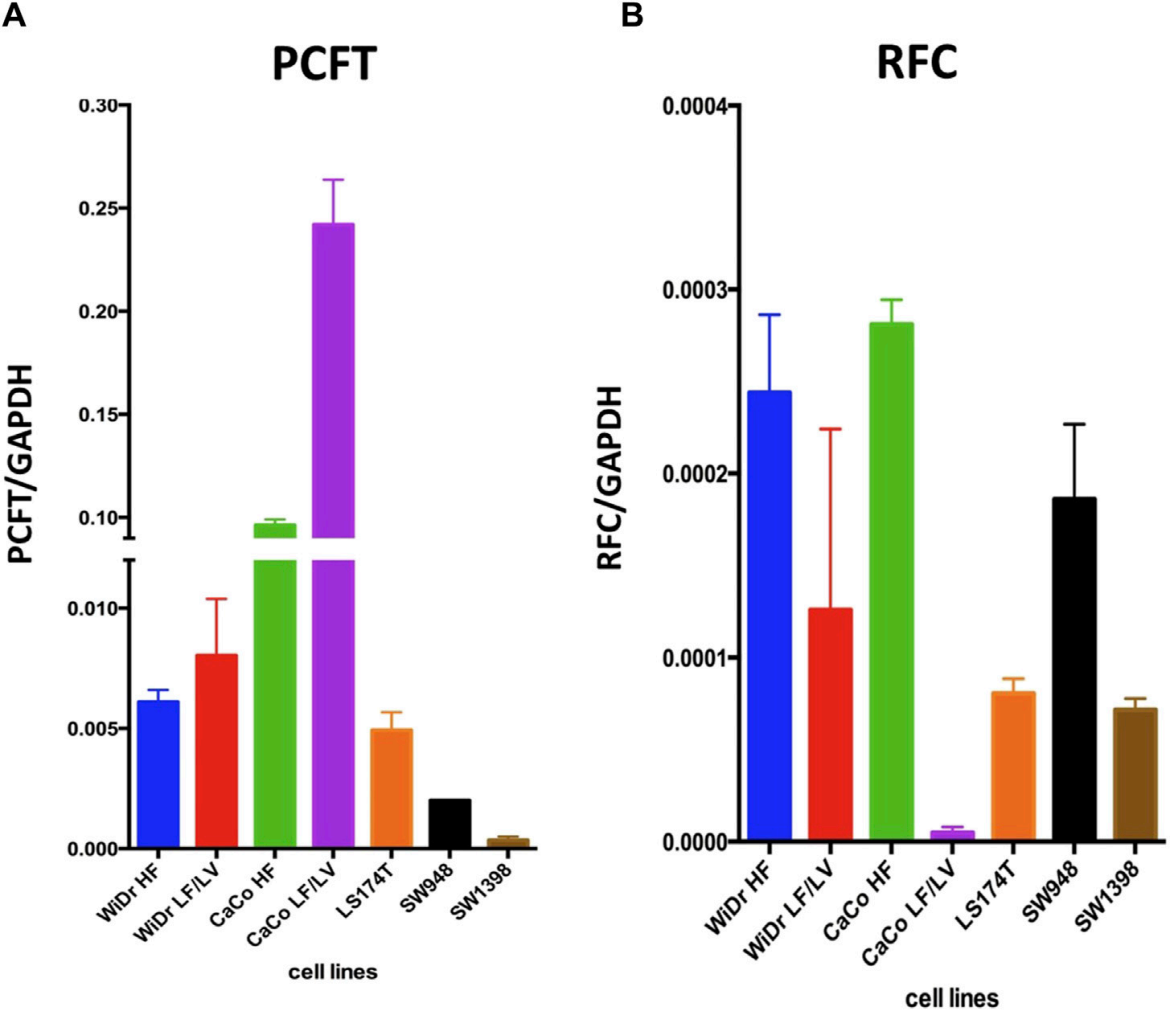
PCFT and RFC mRNA expression in colon cancer cell lines. mRNA expression levels of PCFT **(A)** and RFC **(B)** in colon cancer cell lines as analysed by RT-qPCR. Mean values were calculated from standard curves and expressed relative to the housekeeping gene GAPDH. Data presented are means ± SEM of three separate experiments.

**FIGURE 6:**
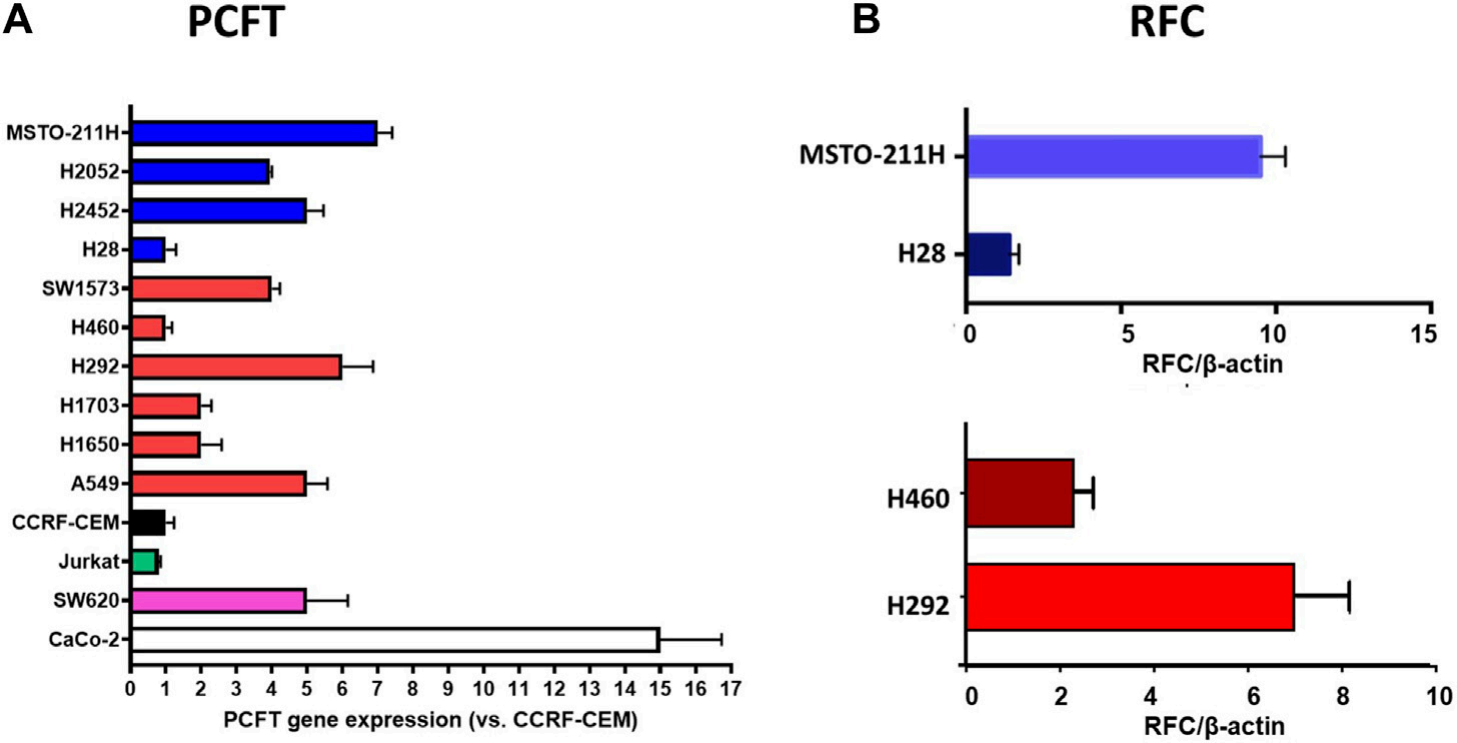
Relative mRNA expression of PCFT and RFC in different types of human cancer cell lines. **(A)** PCFT expression in 14 cell lines, MPM cell lines are depicted in blue, NSCLC cell lines in red. CCRF-CEM (a human acute lymphocytic leukemia (ALL) cell line) is used as a control and set at 1. Other cell lines: Jurkat (CD4 (+) T-cell leukemia), SW620 (colon cancer), CaCo-2 (HF) (colon cancer). **(B)** RFC expression in four selected cell lines Mean values were calculated from standard curves and expressed relative to the housekeeping gene β-actin. Data presented are means ± SEM of three separate experiments.

We next determined the gene expression levels of RFC and PCFT in the cell line panel indicated in Table 1. Results depicted in Figure 5 demonstrate a large difference in the expression of both PCFT and RFC in the panel of colon cancer cell lines. PCFT expression was highest in CaCo2 cells; interestingly PCFT expression was even markedly higher in CaCo2 cells cultured under low folate medium conditions (Figure 5A). Also in WiDr-LF cells, PCFT expression was higher than in wild type WiDr cells cultured under high folate (HF) conditions, but this difference was less pronounced. In three other colon cancer cell lines, PCFT expression was lower than in CaCo2 and WiDr cells. In contrast, RFC was rather constitutively expressed in all colon cancer cell lines (Figure 5B) grown under high folate conditions, except that RFC expression was markedly reduced in CaCo2 cells cultured under low folate conditions and to a lesser extent in WiDr-LF cells. Additionally, PCFT and RFC gene expression was determined in a panel of cell lines of different cancer origin (Figure 6). PCFT expression relative to the reference CCRF-CEM was the highest in CaCo2 cells. From the MPM cell lines, MSTO-211H and H28 showed the highest and lowest expression compared to CEM cells, with values of 7 and 1, respectively. As to the NSCLC cell lines, H292 and H460 cells exhibited the highest and lowest expression relative to CEM cells, with values of 8 and 1, respectively (Figure 6A). Relative RFC expression in MSTO-211H was 7-fold higher than that of H28, while that of H460 was 3-fold higher than for H292 cells.

### 3.3 Modulation by the pure LV stereoisomers and the racemic mixture

#### 3.3.1 Potentiation of 5FU by LV formulations in colon cancer cell lines

**FIGURE 7:**
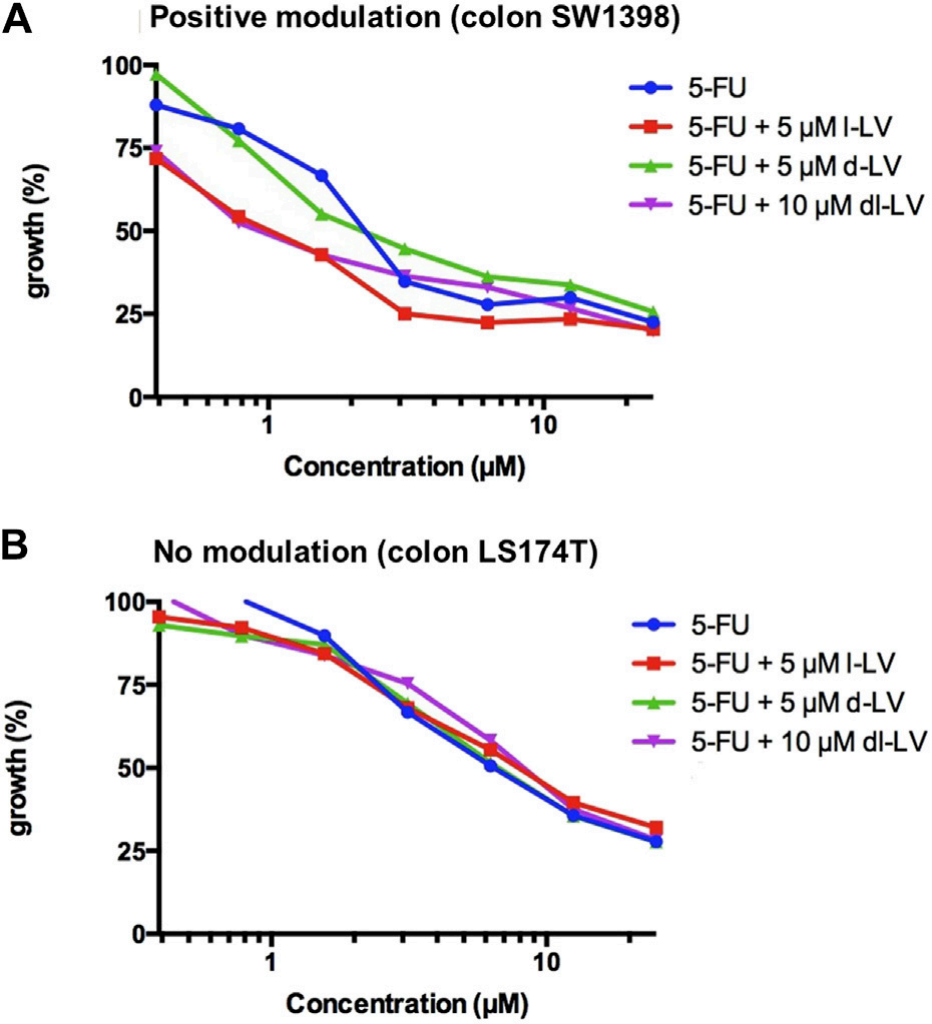
Modulation of 5FU sensitivity by LV-stereoisomers. **(A)** Example of positive modulation in SW1398 cells and **(B)** lack of modulation of 5FU activity in LS174T cells with a comparison of three leucovorin stereoisomer formulations. Representative figure from three separate experiments (SEM was within the size of the marker).

Both, 5-FU alone and 5-FU supplemented with LV (l-, d-, and dl-) caused a concentration-dependent inhibition of growth in all colon cancer cell lines (Figure 7). IC_50_ values were derived from curves as shown in Figure 7, and ranged from 1.8 to 10.5 µM (5-FU only), 1.3 to 8.1 µM (5-FU + 5 µM l-LV), 2 to 10.5 µM (5-FU + 5 µM d-LV), and 1.4 to 8.9 µM (5-FU + 10 µM dl-LV). LS174T was the least sensitive to 5-FU and 5-FU supplemented with LV (l-, d-, or dl-). CaCo2 LF/LV was the most sensitive cell line to 5-FU supplemented with l- and dl-LV (Table 2).

**TABLE 2:** IC_50_ values of 5-FU and 5-FU modulated by 5 μM l-LV, 5 µM d-LV or 10 µM dl-LV for a panel of colon cancer cell lines.

	IC_50_ (µM), 72 h drug exposure			
Cell lines	5-FU	5-FU + 5 µM l-LV	5-FU + 5 µM d-LV	5-FU + 10 µM dl-LV
WiDr	4.56 ± 0.34	1.95 ± 0.20***	4.21 ± 0.57	2.91 ± 1.06**
WiDr LF/LV	1.83 ± 0.18	1.77 ± 0.15	2.78 ± 0.34	1.75 ± 0.08
CaCo2	3.08 ± 0.27	3.04 ± 0.39	3.04 ± 0.66	3.63 ± 0.38
CaCo2 LF/LV	2.05 ± 0.28	1.78 ± 0.18	1.96 ± 0.62	1.78 ± 0.39
LS174T	10.5 ± 0.82	8.11 ± 0.71**	10.5 ± 0.80	8.95 ± 0.72**
HT29	7.90 ± 2.65	3.30 ± 0.20*	7.50 ± 0.50	3.80 ± 0.30*
SW948	3.65 ± 0.41	2.63 ± 0.40***	3.85 ± 0.66	2.78 ± 0.29***
SW1398	2.99 ± 0.47	1.28 ± 0.18***	2.40 ± 0.68	1.44 ± 0.37***

IC_50_ values measured using the SRB assay and are means ± SEM for 3–7 separate experiments. Modulation 5FU growth inhibition by l-LV or dl-LV was significant at the level of ***, *p* < 0.001; **, *p* < 0.01 and *, *p* < 0.05.

Modulation by the various LV formulations varied between the cell lines (Table 2; Supplementary Figure S2). l-LV showed a 2-fold modulation in WiDr, HT29, SW948 and SW1398 cells; however, as shown very clearly in Supplementary Figure S2 d-LV was ineffective in all cell lines in modulating 5FU sensitivity, while in WiDr-LF cells the IC_50_ value for 5FU even increased 1.5-fold by d-LV. The racemic mixture was effective in the same cells where l-LV (after RFC mediated uptake) showed modulation, but the effect was less in WiDr cells (Table 2; Supplementary Figure S2).

#### 3.3.2 Protection of antifolate cytotoxicity by l-LV in lung cancer cells

**FIGURE 8:**
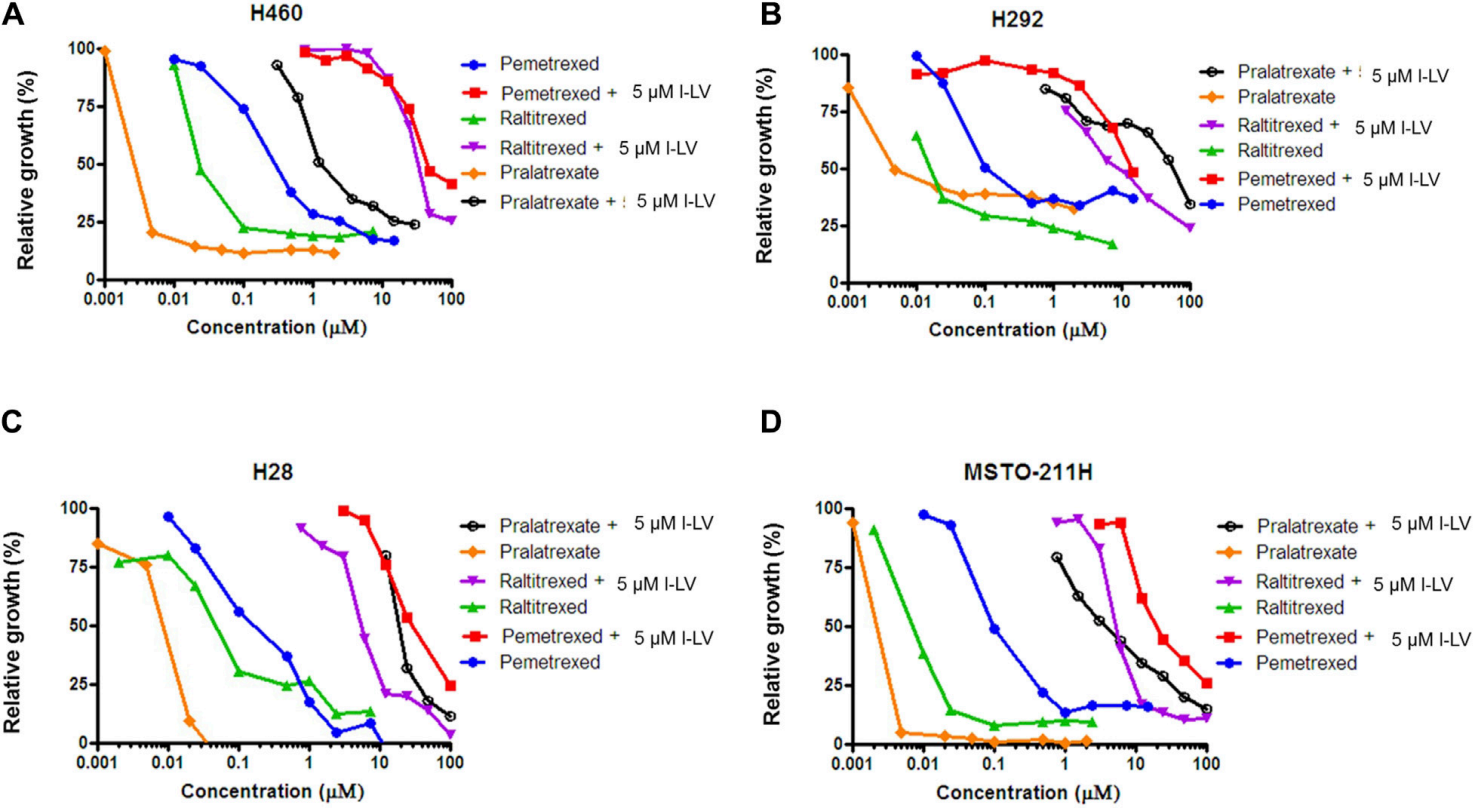
L-leucovorin protection from pralatrexate (PLX), raltitrexed (RTX) and pemetrexed (PMX) growth inhibition of NSCLC **(A,B)** and MPM **(C,D)** cell lines. Dose response curves of growth inhibition of NSCLC cells (H460 and H292) and MPM cells (MSTO-211H and H28) by PLX, RTX and PMX, and protection from growth inhibition by 5 µM l-LV. Representative figures out of three separate experiments are shown. (SEM was within the size of the marker). Drug exposure time: 72 h.

Antifolate sensitivity was determined in four selected NSCLC and MPM cell lines (Figure 8). PMX, PLX and RTX displayed a concentration-dependent inhibition of cell proliferation in all cell lines, with PLX being the most potent antifolate with IC_50_ values ranging from 2.4 nM (MSTO-211H) to 11 nM (H28); RTX displayed intermediate sensitivity with IC_50_ values ranging from 8 nM (MSTO-211H) to 136 nM (H28), whereas PMX was the least active with high IC_50_ values ranging from 103 nM (H292) to 302 nM (H460) (Table 3). Representative growth curves are shown in Figure 8.

**TABLE 3:** IC_50_ values of growth inhibition of NSCLC and MPM cells by pemetrexed (PMX), pralatrexate (PLX) and raltitrexed (RTX) and corresponding protection by l-LV.

	IC_50_ (nM), 72 h drug exposure					
Cell line	PMX	PMX +5 µM l-LV	PLX	PLX +5 µM l-LV	RTX	RTX +5 µM l-LV
H28	280 ± 48	34,333 ± 8,694*	11 ± 0.6	14,600 ± 944*	136 ± 16	4,260 ± 291*
MSTO	143 ± 14	21,000 ± 2,280*	2.4 ± 0.1	3,336 ± 396*	8.0 ± 0.5	4,583 ± 281*
H460	302 ± 12	52,667 ± 8,730*	2.8 ± 0.1	1,450 ± 34*	22 ± 0.9	19,800 ± 2047*
H292	103 ± 12	17,000 ± 141*	3.7 ± 0.3	19,075 ± 6,892*	14 ± 0.8	6,325 ± 662*

IC_50_ values measured using the SRB, assay. Means ± SEM are given. MSTO is MSTO-211H. Statistically significant modulation (*p* < 0.001) is indicated with *.

Protection by l-LV was measured by simultaneous addition of 5 µM l-LV. For all antifolates a large shift in the IC_50_ values was found. The l-LV-mediated increase in the IC_50_ values of PLX was most pronounced (between 502 and 4778-fold) when compared to the other two antifolates, while l-LV protection of PMX cytotoxicity was the least (144-211-fold), possibly related to the intrinsic lower sensitivity of these cells to PMX. Protection by l-LV after RFC mediated uptake was statistically significant (Table 4; Figure 8; Supplementary Figure S3).

**TABLE 4:** Relative protection by l-LV from antifolate (PMX, PLX and RTX) -induced growth inhibition of NSCLC and MPM cells.

	PMX	PLX	RTX
Cell line	Relative protection by 5 µM l-LV		
H28	193 ± 36	1834 ± 108	68 ± 10
MSTO-211H	144 ± 8	1,601 ± 275	592 ± 27
H460	162 ± 41	502 ± 21	900 ± 121
H292	211 ± 43	4,778 ± 1795*	458 ± 46

Values are calculated from the separate ratios of (IC_50_ antifolate + LV/IC_50_ antifolate). Means ± SEM are shown. Protection by l-LV is significant for all three antifolates at the level *p* < 0.0015*.

#### 3.3.3 Efficacy of various antifolates and protection of antifolate cytotoxicity by l-LV in transfected CHO cells; role of PCFT and RFC

In order to determine whether (over)expression of either RFC or PCFT could play a role in protection by l-LV, we used genetically modified cell lines derived from the Chinese hamster ovary (CHO) cell line AA8, specifically expressing either human RFC or human PCFT (Table 1). AA8 cells themselves harbor inherent expression of hamster RFC, but do not express detectable levels of PCFT or FRα. CHO/C5 is a variant in which RFC has been knocked out by site-directed mutagenesis, while CHO/C5 Mock has been mock transfected to serve as a control for CHO/C5 PCFT and CHO/C5 hRFC, which have been transfected with human PCFT or hRFC, respectively.

Parent AA8 cells were clearly most sensitive to each antifolate, with PLX being the most active antifolate, while PMX, RTX and MTX were 10-fold less active (Table 5; Supplementary Figure S3). Protection by l-LV was very efficient, increasing the IC_50_ value from 94-fold (PMX) to 965-fold (PLX) (Table 5). The transporter knock out cells were 83-2500-fold less sensitive to the panel of antifolates than AA8 cells, while the mock transfected cells were 126-3447-fold less sensitive, confirming the absence of any transporter (Supplementary Figure S3). The moderate protection by 5 µM l-LV (Table 5) indicates that at this concentration, passive diffusion may contribute to l-LV uptake. Interestingly, PCFT-transfected cells were similarly sensitive to the antifolates as the knock out and mock transfected cells, both in the absence and presence of l-LV. This indicates that at neutral pH, PCFT is a very poor transporter for these antifolates, being consistent to the transporter experiments shown in Supplementary Figure S1. In contrast, hRFC-transfected cells showed IC_50_ values for the antifolates in the same range as the wild type AA8 cells (Table 5), indicating that under physiological neutral pH conditions, RFC is predominantly responsible for the uptake of antifolates, as well as for l-LV, since protection was very efficient, varying from 72-fold (MTX) to 1462-fold (RTX) (Table 5; Supplementary Figures S3, S4).

**TABLE 5:** Role of various drug transporters in the sensitivity to methotrexate (MTX), pralatrexate (PLX), pemetrexed (PMX) and raltitrexed (RTX) and protection by l-LV/Fusilev^®^ (5 µM).

CHO variants	CHO/C5^a^			MOCK^b^			PCFT^c^			hRFC^d^			AA8^e^		
Drugs	Mean	±	SE	Mean	±	SE	Mean	±	SE	Mean	±	SE	Mean	±	
MTX	8.93	±	1.79	6.67	±	1.36	3.39	±	0.84	0.020	±	0.005	0.038	±	0.001
MTX + l-LV	>100			>100			41.0	±	3.4	1.45	±	0.26	6.60	±	0.31
PLX	9.50	±	0.41	13.1	±	5.6	6.80	±	1.96	0.0038	±	0.0001	0.0038	±	0.0002
PLX + l-LV	48.3	±	8.2	>100			87.5	±	5.31	0.90	±	0.05	3.67	±	0.24
PMX	3.10	±	1.06	42.0	±	3.3	10.6	±	1.5	0.046	±	0.014	0.037	±	0.002
PMX + l-LV	59.6	±	13.3	>100			49.2	±	8.5	29.3	±	6.4	3.50	±	0.64
RTX	2.73	±	0.42	2.4	±	0.4	4.33	±	0.2	0.008	±	0.001	0.019	±	0.001
RTX + l-LV	6.68	±	0.70	>100			52.6	±	9.7	11.7	±	2.3	2.92	±	0.51

IC_50_ (in µM) values were determined at least three times and are shown as means ± SEM for each cell line and antifolate drug. l-LV was added at 5 µM concentration.

^a^ CHO/C5: RFC-deficient cell line.

^b^ CHO/C5-MOCK: empty vector transfected cell line.

^c^ CHO/C5-PCFT: PCFT-transfected cell line.

^d^ CHO/C5-hRFC: human RFC transfected cell line.

^e^ AA8: parental CHO cell line. For all CHO variants protection by l-LV was significant at the level <0.001.

### 3.4 Potentiation by l-LV of the cellular effects of 5FU and protection against antifolates

**FIGURE 9:**
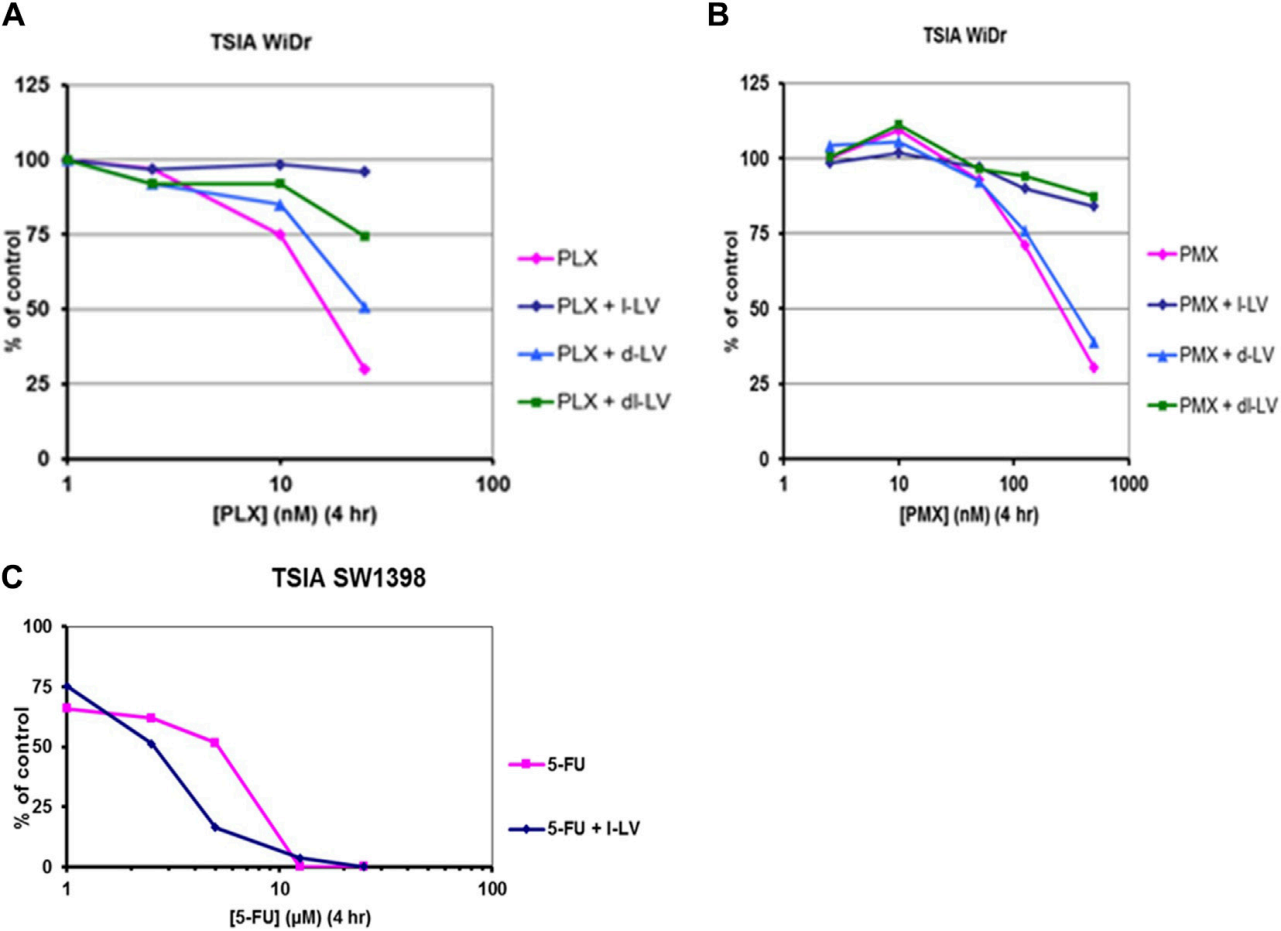
Antifolate- and 5FU-inhibition of *in situ* TS activity (TSIA assay) in colon cancer cells and protective/potentiating potential of LV-stereoisomers. Concentration-dependent inhibition *in situ* TS activity in WiDr cells by **(A)** PLX, and **(B)** PMX in the absence or presence of 5 μM l-LV, 5 µM d-LV or 10 µM dl-LV. **(C)**: concentration-dependent potentiation of inhibition of *in situ* TS activity in SW1398 cells by 5FU in the absence or presence of 5 µM l-LV. Exposure time to the drugs was 4 h, with [5-^3^H]-deoxycytidine present for the last 2 h. Representative curves out of three separate experiments are shown. SEM was within the size of the marker.

Another approach to investigate the functionality of the antifolates and the modulation of 5FU efficacy by LV is using the thymidylate synthase *in situ* inhibition assay (TSIA) (Figure 9). This assay measures the intracellular inhibition of TS, and incorporates all limiting intracellular factors necessary to achieve this inhibition, such as uptake and metabolism (e.g., conversion to polyglutamate forms).

In order to study the effect of the various LV formulations on the potentiation of 5FU-mediated inhibition of TS, we used several colon cancer cell lines. (Table 6; Figure 9). The data reveal that TS inhibition dynamics mimicked growth inhibition profiles; inhibition of TS is achieved quite fast in sensitive WiDr cells, whereas slow TS inhibition was observed in the least sensitive LS174T cells. In this cell line inhibition of intracellular TS could be enhanced moderately by l-LV and dl-LV but not by d-LV. The other three colon cancer cell lines were much more sensitive to 5FU itself and TS inhibition could be modulated by all three LV formulations, including d-LV, but to a lesser extent. Interestingly, with the least sensitive cell line, LS174T, potentiation by dl-LV was more pronounced than with l-LV alone.

**TABLE 6:** TSIA-IC_50_ values for 5FU, PLX, RTX, and PMX and stimulation or protection by different leucovorin formulations in colon cancer cells.

Cell line	Drugs	+ l-LV	+ d-LV	+ dl-LV
	5FU (μM)	5FU	5FU	5FU
WiDr	3.76 ± 0.35	2.35 ± 0.20***	3.00 ± 0.54	2.34 ± 0.25**
WiDr-LF	3.67 ± 0.33	2.37 ± 0.30**	2.77 ± 0.24**	2.70 ± 0.23**
SW1398	4.14 ± 0.24	2.52 ± 0.28***	3.28 ± 0.23**	2.45 ± 0.27***
LS174T	60 ± 20	48 ± 15	60 ± 5	36 ± 3.7*
	PLX (nM)	PLX	PLX	PLX
WiDr	17.7 ± 4.7	>100***	17.0 ± 1.4	>100***
	RTX (nM)	RTX	PLX	PLX
WiDr	32 ± 12	>1,000***	37 ± 14	>1,000***
	PMX (nM)	PMX	PMX	PMX
WiDr	175 ± 75	>1,000***	260 ± 60	>1,000***

Values are means ± SEM of three separate experiments performed in duplicate. Cells were exposed for 4 h to the drugs in a concentration range, while the leucovorin (LV) formulations were added simultaneously. Concentrations of 5FU are given in µM, those for the antifolates are given in nM. The concentrations were 5 μM l-LV, 5 µM d-LV, or 10 µM dl-LV. TSIA-IC_50_ values (50% inhibition) were determined from separate curves. TSIA modulation by LV variants was significant at the level of ***, *p* < 0.001; **, *p* < 0.01; *, *p* < 0.05.

Since the assay was validated with WiDr cells, this cell line was also used to investigate the effect of these formulations on protection against antifolates. Earlier WiDr cells were also characterized for sensitivity to antifolates with similar values in the low nanomolar range (Peters et al., 1999; Van Triest et al., 1999). The differences in modulation of TS inhibition were much more pronounced for the antifolates. Although we used a relatively short 4 h drug incubation, inhibition of intracellular TS was achieved in the nanomolar range (17–175 nM). PLX was the most potent drug, possibly because it has a highly efficient RFC uptake and conversion to polyglutamate forms that inhibit both DHFR and TS (Raz et al., 2016).

In contrast, PMX uptake by RFC is less efficient, as is its conversion to polyglutamate forms in 4 h, thus resulting in less potent TS inhibition. 5 μM d-LV does not display any protective effect on the TSIA (Table 6). The TSIA IC_50_ values are comparable to those of the antifolates alone. In full contrast, protection by 5 µM l-LV of TSIA was very efficient for all three antifolates, leading to dramatic shifts (>100–1000-fold) in TSIA IC_50_ values when compared to conditions without LV (Table 6). Moreover, dl-LV was similarly effective as l-LV in preventing the antifolates to inhibit the cellular TS activity.

## 4 Discussion

In the current study we show that the expression of the major folate transporters RFC and PCFT plays an important role in the modulatory effect of the active form of LV, l-LV. At physiological pH, both l-LV and dl-LV enhance the cytotoxicity of 5FU against colorectal cancer cells, while the functional effect using the TSIA assay is even more pronounced. This functional effect is even more evident regarding the protection exerted by LV formulations on cytotoxicity of the three antifolates PMX, RTX and PLX against NSCLC and MPM cell lines. l-LV completely protects against the cytotoxicity of these antifolates including RTX and PLX which are excellent transport substrates for RFC, but also for PMX, which is a transport substrate for both RFC and PCFT. However, the data with the transfected cell lines clearly demonstrate that at physiological pH, PCFT does not play any role in the cytotoxicity of PMX.

This investigation regarding the comparison of l-LV and d-LV was initiated because in some papers it was questioned which folate would be most optimal for either enhancing the effect of 5FU or to be used as a protective agent against toxic side effects of commonly used antifolates (Chuang and Suno, 2012; Hayes et al., 2014; Maruvada et al., 2020). Regarding modulation of 5FU, we used several CRC cell lines, previously characterized for their sensitivity to 5FU and the modulation by dl-LV (Van Triest et al., 1999). In a large panel of unselected cell lines with a different pathology, dl-LV enhanced the effect of 5FU about 2-fold (Van der Wilt and Peters, 1994). In general, modulation by l-LV is similar to dl-LV, while d-LV does not modulate, in agreement with data in a CCRF-CEM cell line (Zittoun et al., 1991). In the present CRC cell line panel, modulation is also about 2-fold which is obviously related to the efficient uptake of reduced folates by these CRC cells (Van Triest et al., 1999); uptake will not be affected by d-LV since this compound has a poor affinity for RFC compared to l-LV (Figure 3). Interestingly, the data of the functional assay (the TSIA) allowed to quantify the role of folate uptake and metabolism, clearly indicating the importance of RFC in cellular uptake of reduced folates and antifolates in cancer cells with a high variation in all transporters. One important point of the functional assay not described earlier was the moderate but significant effect of d-LV on 5FU induced TS inhibition. This can be explained by the stimulating effect of d-LV on the binding of FdUMP on TS (Van der Wilt et al., 1993). Regarding l-LV and other naturally occurring S stereoisomers of reduced folates, not only the monoglutamate forms stimulate FdUMP binding to TS, but polyglutamylated forms are even more efficient (Van der Wilt et al., 2002). However, we do not expect that a polyglutamated form of d-LV will mediate this effect. To the best of our knowledge d-LV cannot be polyglutamylated or very poorly, since d-forms are poor substrates for FPGS (McGuire et al., 1980), while uptake will be low because of its poor substrate characteristics (Figure 3) and mainly mediated by passive diffusion. Previously we also demonstrated that a high expression of FPGS is important for the modulation of 5FU by LV (Van Triest et al., 1999). Indeed, clinical studies also showed a relation of 5FU-LV efficacy with FPGS expression (Chazal et al., 1997; Cheradame et al., 1997). Our data are in agreement with earlier papers on transport of-l-LV and dl-LV (Sirotnak et al., 1979; Matherly and Huo, 2008; Zhao et al., 2009). Since l-LV is one of the best transport substrates for both RFC and PCFT, a high expression of these transporters, both in tumor cells and normal cells, enables a fast and efficient uptake of l-LV. However, the high expression of PCFT in NSCLC and MPM plays an important role in the sensitivity of these malignancies to PMX (Zhao et al., 2009; Giovannetti et al., 2017; Matherly et al., 2018), which is an excellent substrate for PCFT at a low pH. The role of PCFT for PMX uptake is enhanced under acidic conditions, such as the tumor micro-environment of NSCLC and MPM. Indeed a high PCFT expression, but not RFC, was related to efficacy of PMX in mesothelioma (Giovannetti et al., 2017). Although RFC also has a high expression in NSCLC and MPM cells and tissues (Giovannetti et al., 2008; 2017), it does not play a role in the antitumor activity of PMX, possibly because in the tumor microenvironment, the pH is relatively low and RFC will be inactive.

Normal tissues such as gut epithelium, which has a relatively low pH in the duodenum and is more acidic at the bottom of crypt cells (Amiri et al., 2021), a high PCFT will enable uptake of l-LV. The colon epithelial cancer cell line CaCo2 has a high PCFT expression and is widely used as a model for intestinal drug uptake. Caco2 forms a polarized structure in transwell systems (Honeywell et al., 2015), displaying the natural distribution of transporters in gut epithelium (with PCFT at the apical gut site). Previously we observed a high uptake under these conditions of both folic acid and MTX (Lemos et al., 2007). Both compounds are relatively good substrates of PCFT, which explains the important role of PCFT as folate transporter in the gut. This also explains the efficacy of folic acid in protecting normal tissues (including gut) against antifolates (MTX and PMX) side effects. However, the gut has also a high FRα (Nutt et al., 2010), enabling a relatively efficient uptake of folic acid, commonly used to control toxicity in combination treatment with PMX in malignancies (Vogelzang et al., 2003; Nutt et al., 2010) and with MTX in rheumatoid arthritis (van Ede et al., 2001). The latter may be a problem when RA patients do not only take a low dose of folic acid in combination with low dose MTX, but also food fortification formulas (which contain a high dose of folic acid) which might completely abolish the efficacy of MTX (Maruvada et al., 2020).

LV is usually given as an infusion, but oral administration is also feasible (Priest et al., 1991). Although d-LV might affect the uptake of l-LV, this effect may be limited since d-LV has a much higher Kd for FRα compared to l-LV (Wang et al., 1992), which is in agreement with our data. However, d-LV may also affect l-LV pharmacokinetics when given as an infusion of the racemic mixture (Priest et al., 1991), although others did not find an interaction (Bertrand and Jolivet, 1989; Zittoun et al., 1993). However, this might be related to the less specific assays in these papers. Differences in pharmacokinetics and metabolism may be bypassed when another folate is administered to modulate 5FU, which can be achieved by administration of 5,10-CH_2_-THF itself, which is feasible when a proper formulation is used (Glimelius et al., 2021). Initially, Modufolin was used for this purpose (Danenberg et al., 2016), but large clinical studies were feasible with a more stable formulation of 5,10-CH_2_-THF (Arfolitixorin). The efficacy of the FOLFOX schedule was comparable to a schedule in which dl-LV was replaced by Arfolitixorin, although the study was designed to find a higher efficacy of the Arfolitixorin arm (Tabernero et al., 2024). However, in that schedule the effect of 5FU modulation might be overruled by the addition of oxaliplatin, since platinum analogs may decrease thymidylate synthase as well (Van der Wilt et al., 1992a).

This study also shows that mechanisms of folate transport and metabolism can efficiently be studied when using adequate *in vitro* models, in contrast to most *in vivo* models. Especially for (anti) folates both mice and rats are not appropriate, since the standard chow for these animals is extremely rich in folates (mostly oxidized and 5-methyl-tetrahydrofolate). However, these folates do not affect the modulation of 5FU by LV in mice, since both l-LV and dl-LV have a similar potentiating effect (Van der Wilt et al., 1992b). However, oxidized folates and 5-methyl-tetrahydrofolate will efficiently neutralize the antitumor effect of DHFR inhibitors, such as MTX which is ineffective in mouse models (Braakhuis et al., 1985). This can, partially, be overcome by putting the animals on a folate free diet and supplement this with a low amount of folic acid. The latter will protect normal tissues, but not the tumor (Smith et al., 1995; Alati et al., 1996; Van der Wilt et al., 2001). Next to folates, mice have a high thymidine level in plasma. Thymidine will also efficiently protect against antifolates including TS inhibitors such as RTX and PMX (Smith et al., 1995; Van der Wilt et al., 2001). The effect of thymidine can be prevented by either using thymidine kinase deficient models or by supplying thymidine phosphorylase (either pegylated or as pure enzyme) which will breakdown thymidine to undetectable levels (Direcks et al., 2008; Honeywell et al., 2015, unpublished data). Alternatively, in order to study side effects of MTX, adequate *in vitro* models such as a clonogenic assay for bone marrow toxicity (Jolivet et al., 1994) can be used. Moreover, oral mucosa organoids have been shown to be excellent *in vitro* models to study MTX induced mucosal toxicity (Driehuis et al., 2020).

Our study shows that l-LV is an excellent substrate for both RFC and PCFT, in agreement with earlier studies. However, this study is the first that systematically compared the various LV forms (l-LV, d-LV and the racemic mixture) for their potential capacity to modulate 5FU cytotoxicity and protect cells against the clinically used antifolates PMX, PLX, RTX and MTX. Modulation of 5FU can be achieved very effectively by l-LV, although d-LV itself may potentiate the binding of FdUMP to thymidylate synthase. At physiological pH, l-LV, taken up by RFC, was very effective in protecting cells against antifolates. This provides an additional perspective using l-LV as a fast protective or rescue agent (taken up by RFC) against toxicity induced by these antifolates, especially for myeloid cells growing at physiological non-acidic conditions. The low pH of the tumor microenvironment of NSCLC and MPM enables the effective therapy for PMX, one of the best transport substrates of PCFT. However, normal cells at a physiological pH (around 7) can be selectively protected by RFC-mediated uptake of systemic l-LV, which has a rapid distribution.

## Data Availability

The original contributions presented in the study are included in the Article/Supplementary Material, further inquiries can be directed to the corresponding author.

## Supplementary material

SUPPLEMENTARY FIGURE S1 Differential inhibition of PCFT-mediated influx of [^3^H]-l-LV (2.5 µM) at pH 5.5 and 7.4 by equimolar concentrations of l-LV, d-LV, dl-LV, PMX and PLX. PCFT influx activity of [^3^H]-l-LV at pH 7.4 was 11.5% of that at pH 5.5, calculated from the separate activities measured in each experiment. Values are means ± SEM of at least three separate experiments.

SUPPLEMENTARY FIGURE S2 Modulation of 5FU cytotoxicity by three different leucovorin formulations in colon cancer cells. Modulation effect is expressed as the ratio of the mean IC_50_s for 5FU in the presence of LV stereoisomer (l-LV, d-LV or dl-LV) divided by the mean IC_50_s for 5FU in the absence of LV. Drug incubation time: 72 h. No modulation is represented by the red line crossing 1.0. Since values are calculated from the means depicted in Table 2, no SEM could be shown. However, the table shows the SEM for each condition.

SUPPLEMENTARY FIGURE S3 Relative sensitivity for MTX, PLX, PMX and RTX of AA8 and of PCFT and hRFC transfected CHO/C5 cells compared to CHO/C5 cells without hRFC/PCFT transporters. The ratio is calculated by dividing the mean IC_50_ of CHO/C5 cells by those of AA8 and the transfected panel. Relative sensitivity to CHO/C5 is equal to 1, represented by the black line. The ratio represents the mean fold decrease in IC_50_ values, which corresponds to the fold increase in sensitivity. Since values are calculated from the means depicted in Table 5, no SEM could be shown. However, the table shows the SEM for each condition.

SUPPLEMENTARY FIGURE S4 Fold protection of the cytotoxicity of MTX, PLX, PMX, RTX by l-LV in AA8 cells, CHO/C5 and PCFT- and hRFC-transfected CHO/C5 cells. The ratio is calculated by dividing the mean IC_50_ value in the presence of l-LV (5 µM) by that without l-LV. When a complete protection was found, the highest concentration tested for the drug was used for the calculation. The ratio thus represents the fold increase in IC_50_, or the fold protection. Since the values are calculated from the means no SEM can be depicted. However, the table shows the SEM for each condition.
