# Supplementary figures and images for: The role of l-leucovorin uptake and metabolism in the modulation of 5-fluorouracil efficacy and antifolate toxicity
Source: Front Pharmacol. 2024 Aug 21;15:1450418. doi: 10.3389/fphar.2024.1450418 (PMC11371747; doi:10.3389/fphar.2024.1450418)

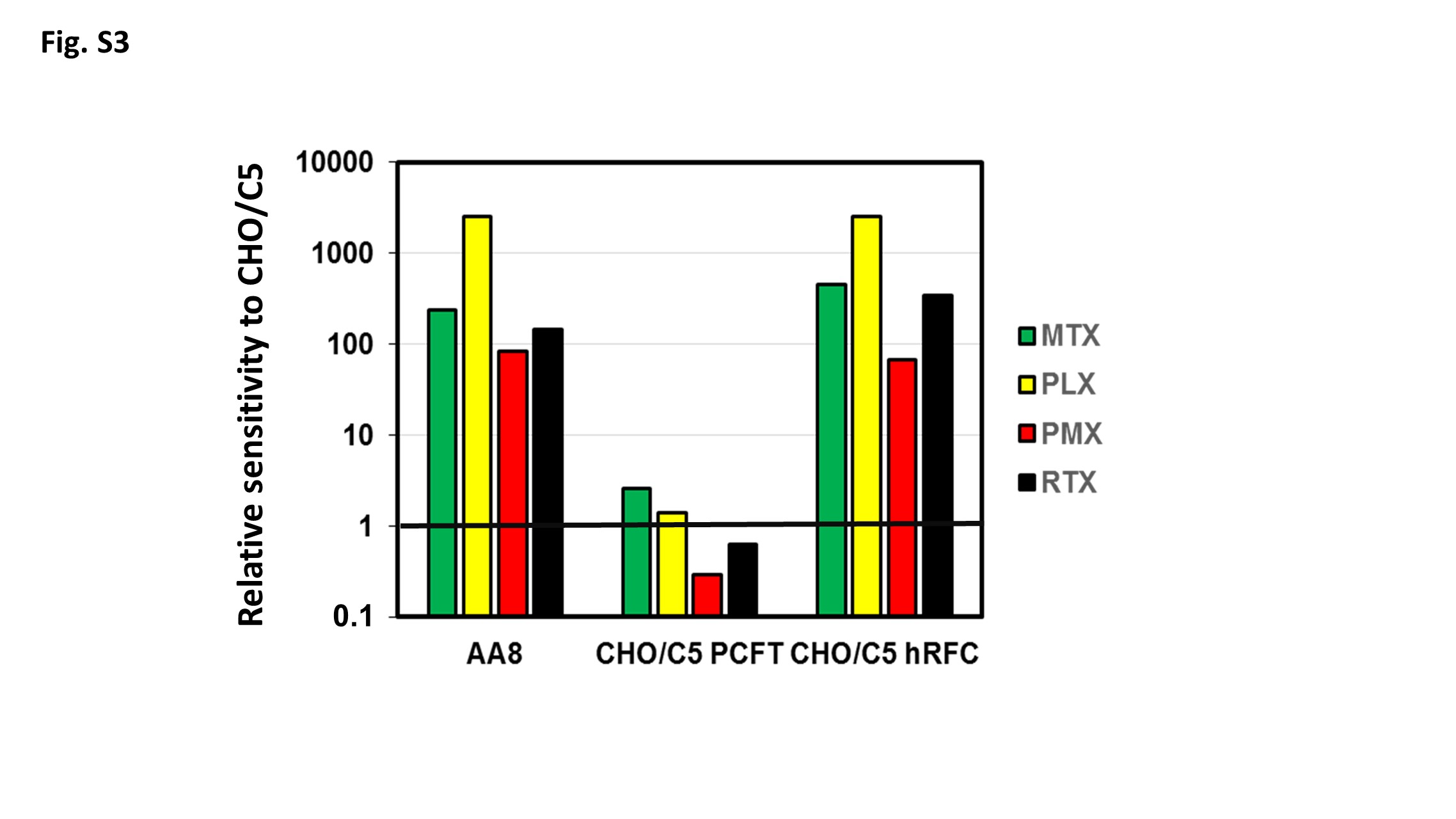

Supplement: Supplementary file 1 [file Image3.JPEG]

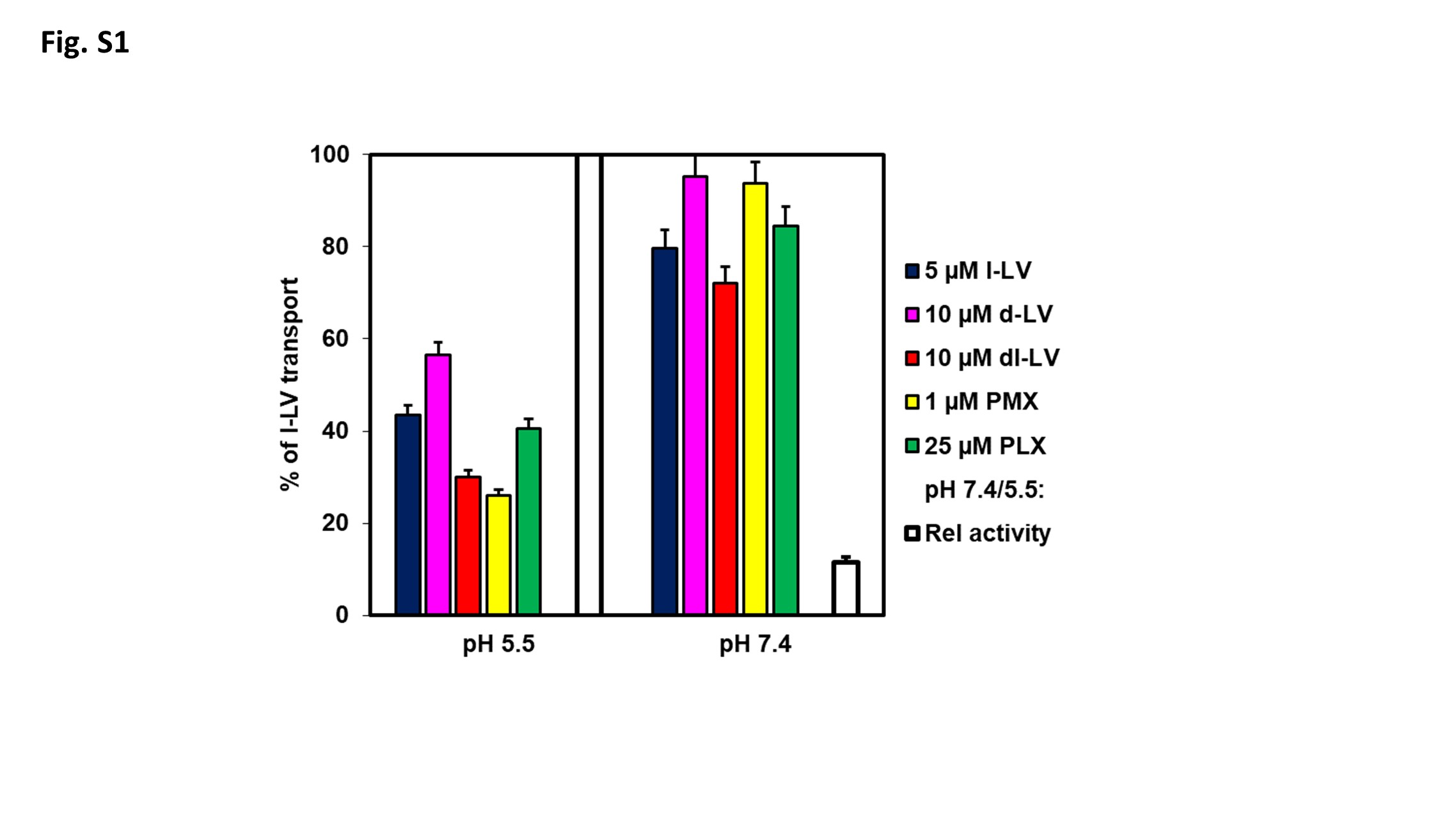

Supplement: Supplementary file 2 [file Image1.JPEG]

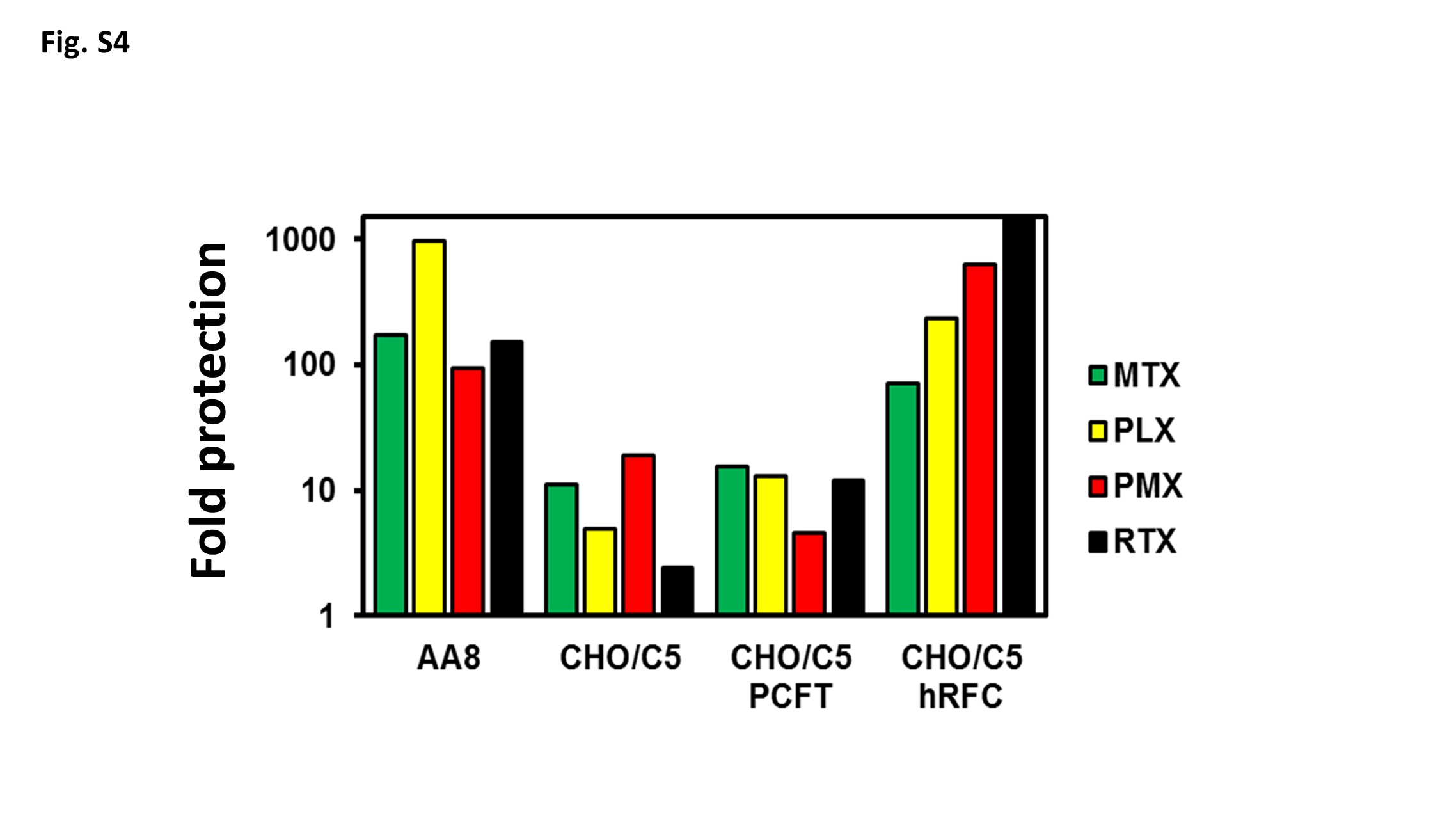

Supplement: Supplementary file 3 [file Image4.JPEG]

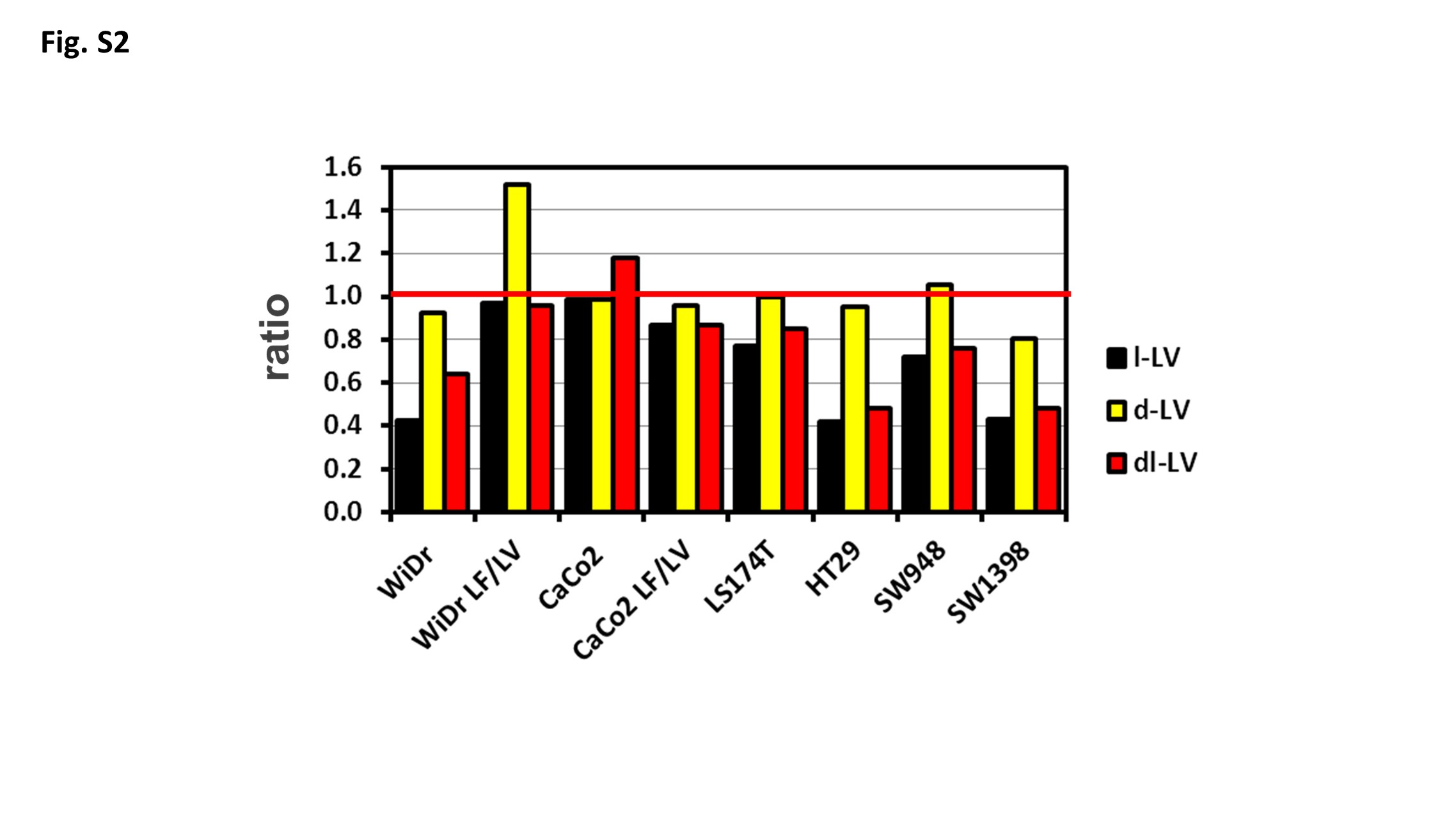

Supplement: Supplementary file 4 [file Image2.JPEG]
